# St. George's Hospital

**Published:** 1830-06

**Authors:** 


### ST. GEORGE'S HOSPITAL.

### *Compound Fracture of the Leg, accompanied by Delirium Tramaticum, treated with large Doses of Opium.**

J. R., forty-five years of age, was admitted into St. George's Hospital on the evening of the 6th of November last, under the care of Mr. Keate, with fracture of both bones of the left leg. The tibia was broken just below its centre, and badly comminuted. The compresses were soaked with blood, which had escaped from a small wound situated over the fracture, and now scarcely perceptible. The skin in the neighbourhood was thin and dark coloured, with several small vesications. The fracture of the fibula was at its upper part. The whole limb was swelled to a large size, partly from effused blood, but chiefly from œdema. The opposite leg was also œdematous.

The accident happened on the preceding evening, a few miles from town, and was produced by his attempting, when in a state of intoxication, to get on a coach, which was moving at a rapid pace. One of the wheels is said to have struck the limb, but not to have passed over it.

His general appearance was very unfavorable, the complexion being sallow, interspersed with spots of acne rosacea: his sharp sunken eye, with the arcus senilis, his scanty grey hairs, and trembling hand, bespoke premature old age. He must once have

* Medical Gazette.

been a stout man, but was now thin, and of a relaxed fibre. He stated himself to have been a wine and spirit merchant, in respectable circumstances, and confessed that he was addicted to the immoderate use of both spirituous and fermented liquors; that he had been occasionally the subject of gout; and, judging from his own account, had once or twice suffered from symptoms resembling delirium tremens; notwithstanding which, he described his general health as being tolerably good. Since the accident, he had suffered from troublesome diarrhœa, and had had no sleep. The pulse was eighty-six and steady; skin cool; tongue furred in the centre, but moist; leg not very painful. Limb placed in a junk, to be kept wet with a strong camphorated spirit lotion; and he was ordered Liq. Opii Sedativ. ♏l. ex haust. Camph. ℥iss. statim.

In two hours after, being restless, the draught was repeated, with ♏xxv. of the Liq. Opii.

8th.—Limb going on favorably. The wound has healed, and the appearance of the skin has greatly improved; the diarrhœa has ceased. He has been taking the following draught every six hours: Liq. Opii Sedat. ♏xxx.; Ammon. Carbon. gr. vj.; Mist. Camph. ℥iss. His nourishment has consisted of beef-tea, arrowroot, &c. Has had little or no sleep since his admission.—To repeat the draught, with an increase of ♏xx. of the Liq. Opii at bedtime, and to have ℥iij. of gin daily.

9th.—Last night he became very restless, tossing about in and attempting to get out of bed, and talking much of law business in which he was concerned; but, when spoken to, he answered rationally. The pulse became less steady in its beat, and quicker. Some strong gin and water was given him, and a double dose of opium. He slept a little towards morning, but even then he kept constantly moving the limb about in bed. He is today much more composed, but quick and irritable in his manner. Pulse upwards of 100; tongue furred; bowels confined.—The limb to be placed in Amesbury's apparatus. To repeat the draught of Liq. Opii Sed., with a drachm dose at bedtime, and to have six ounces of gin daily.

10th.—Another restless night, and no sleep; the bowels have acted freely from opening medicine. He states that he has been in the habit, when not well, of occasionally taking the Tinct. Opii in drachm doses.—To continue the draught every six hours, with a drachm of Liq. Opii Sed. in each.

11th.—Last night the delirium and restlessness greatly increased, and, in the absence of the nurse, he got out of bed and began to dress himself. On being spoken to, his answers were quite rational, although his manner was extremely quick and irritable. Pulse today ninety-six, of sufficient strength; bowels open; tongue clean; leg looking well: the bones are kept in good apposition by the apparatus, in spite of his constant motion.

12th.—Last night the delirium, &c. became worse than ever, and he was with difficulty kept in bed. At eleven P.M. three grains of the Ext. Opii were given him, and repeated at intervals of two or three hours. During the night he took nine grains of opium, which did not procure him any sleep. Today the pulse is 100; tongue a little furred; the leg more swelled.—To discontinue the six-hour draught, and to take every two hours three grains of the Ext. Opii.

Nine P.M.—Having taken two doses without any effect, four grains of the powdered opium, with a quarter of a grain of Ant. Tart. were administered, and one pound of porter was given him; soon after which he fell asleep, and slept soundly for several hours, during which time both hands and legs were observed to be constantly in motion, and he sometimes struck one side of the face with one hand, and the other side with the other, for a minute together without awakening, and made other odd gestures.

13th.—Last night, at ten P.M., he took one five-grain dose of Pulv. Opii; soon after which he fell asleep, and slept soundly the whole night. He is today much better, but very irritable. Pulse 100; tongue furred; bowels confined; leg doing well, but of large size; fluctuation of fluid to be felt over the fracture.—To continue the gin, porter, &c.

14th, eight A.M.—Since yesterday he has taken twelve grains of Pulv. Opii, but has not once slept, and passed a very restless night; but is in other respects the same.—To have Oss. gin daily, and to take the opium in seven-grain doses.

15th.—Up to ten o'clock last night, he had taken during the day twenty-eight grains of Pulv. Opii; was now given a ten-grain dose, but he passed a sleepless night, although quieter than the last. Today, however, he is very restless, with constant starting of the limb, which he says is produced by some one striking him violently on the calf of the leg, with a stick or stone. Talks sensibly, but wanders at times. Pulse ninety; tongue furred; bowels open.—It was now directed that he should take one grain of the Acetate of Morphia every two hours. The stimuli to be continued.

16th, ten A.M.—Since yesterday morning he has taken nine grains of the morphia, without any effect, his night having been worse than any since his admission; the delirium, &c. being constant. The leg is doing tolerably well, although the bones have been a little displaced by his jumping out of bed; fluid over the fracture becoming absorbed.—Rep. gin, porter, &c.

N.B. The acetate of morphia is known to be good, having been procured at Mr. Garden's, in Oxford street.

17th.—Up to six o'clock last evening, he had taken a scruple of powdered opium, and a drachm of the Tinct. Opii had been given by the rectum, but he remained as restless as ever. He now took a scruple dose of crude opium, soon after which he fell asleep, and continued to sleep soundly for five or six hours. Today he is quite a different man, being much less irritable, &c. Pulse ninety, wanting power; bowels open. The fluid over the fracture has become absorbed. The leg is doing well, but becomes so much swelled towards night as to require all the straps of the apparatus to be loosened.—To take the gin as usual, with ℔ij. of porter, and meat diet.

18th.—Towards evening delirium came on, with great restlessness. A scruple of crude opium was given him, but not producing any effect, ten grains more were administered; after which he slept soundly for many hours, and is today better than he has ever been since his admission.

19th.—Took a scruple of crude opium last night in two doses, and slept well. Leg going on favorably; much less starting of the limb.

20th.—An attempt was made last night to reduce the dose of opium to fourteen grains, but he became very restless, and it was found necessary to give half a scruple more; after which he slept tolerably well, but is more irritable in his manner today.

22d.—For the last two nights he has taken each night a scruple of the crude opium, and has rested well. This has always been given in two doses, the second not being given till his restlessness, &c. required that it should be administered. Leg examined, and union found to be very considerably advanced.

24th.—Not quite so well, and last night he took half a drachm of the crude opium before sleep could be produced. He yesterday began to complain of pain and stiffness about the right shoulder, but nothing can be discovered in this situation to account for it: thinks himself that it is rheumatic.

27th.—Since the last report he has continued to take a scruple dose of crude opium at bedtime, combined with a scruple of P. Ipecac. comp. The delirium has been very slight, but no sleep could be procured without the opiate. He still complains of the pain in the shoulder, which is now swelled and somewhat tender, but moving the arm does not produce pain; has had no rigors.

29th.—The tumor in front of the shoulder-joint has increased to a large size, and fluctuation is distinct. No rigors, but considerable febrile disturbance. Has taken no opium since the evening of the 27th: no delirium; has rested tolerably well; leg going on as well as possible.—Ordered Bark, with the ammoniated Tincture of Bark, every six hours. To take Oss. of red wine daily, with the porter and fish diet. Discontinued the gin.

December 1st.—Abscess punctured, and a large quantity of well-formed pus evacuated, which did not in anywise communicate with the joint, but a probe passed to the under surface of the scapula.—A poultice to the shoulder.

15th.—There has been no return of delirium, and he has only occasionally taken a small dose of Tinct. Opii at bedtime. The discharge from the abscess has greatly diminished. The apparatus was removed from the leg ten days back, the bones having become well and firmly united; common splints applied. General health much improved. He continues to take the bark, wine, porter, &c.

On the 18th of January, the abscess having been healed up for some days, and the leg being quite strong, he was discharged cured.

N.B. During the period at which he was taking the opium, the bowels were not more confined than they would probably otherwise have been, and were easily acted on by half an ounce of Haust. Sennæ. On leaving off the opium he had a diarrhœa, which was restrained by the usual remedies.

